# Validation of the revised Diabetes Self-Management Questionnaire (DSMQ-R) in the primary care setting

**DOI:** 10.1186/s12875-021-01615-5

**Published:** 2022-01-11

**Authors:** Bernadett Márkus, Csenge Hargittay, Barbara Iller, József Rinfel, Péter Bencsik, Ilona Oláh, László Kalabay, Krisztián Vörös

**Affiliations:** 1 Department of Family Medicine, Semmelweis University, Stáhly str. 7-9, Budapest, 1085 Hungary; 2 Department of Primary Health Care, University of Pécs, Rákóczi str. 2, Pécs, 7623 Hungary; 3 Independent researcher, Budapest, Hungary; 4 Tóth Ilona Health Service Clinic, Görgey Artúr square 8, Budapest, 1212 Hungary; 5 Department of Internal Medicine and Hematology, Semmelweis University, Szentkirályi str. 46, Budapest, 1088 Hungary

**Keywords:** Diabetes / Insulin Resistance, Lifestyle Modification/ Health Behavior Change, Patient Education, Primary Care, Quality of Life, Self-management

## Abstract

**Background:**

Available tools measuring self-management in diabetes are often improperly validated or do not correlate with glucose metabolism. The Diabetes Self-Management Questionnaire (DSMQ-R) is a valid tool, that showed strong relationship with glucose metabolism in tertiary care among people with mostly type 1 diabetes.

**Aim of the study:**

To validate the translated DSMQ-R questionnaire in a Hungarian sample of people with predominantly type 2 diabetes in primary care.

**Methods:**

We enrolled 492 adults from 38 practices in this cross-sectional cohort study, who filled out the self-administered questionnaire, consisting of DSMQ-R and the Summary of Diabetes Self-Care Activities (SDSCA) questionnaires. Family doctors provided clinical data. The translation process was performed in six steps, reaching the expert committee appraisal. The validity of the questionnaire was evaluated by assessing reliability and construct validity.

**Results:**

Cronbach’s alpha showed the questionnaire to reach good reliability (α = 0.845), although subscales had lower alphas. Contrary to the SDSCA questionnaire, the DSMQ-R sum scale differed significantly between persons on target vs not on target (median (interquartile range): 7.23 (6.17–8.44) vs 6.91 (5.91–8.02), and the DSMQ-R sum scale correlated significantly with BMI, HbA1c and SDSCA sum scale. In multivariate analysis higher DSMQ-R scores were significant predictor of achieving glycemic target goal.

**Conclusion:**

The Hungarian translation of the DSMQ-R is a comprehensible tool to assess self-management of persons with diabetes. The questionnaire is valid and reliable in family practice, although its association with achievement of diabetes HbA1c target is weaker in primary than in tertiary care.

## Background

The global prevalence of diabetes mellitus has reached 9.0% in adult men and 7.9% in women in 2014 [1]. In Hungary 7.27% of the population turns in antidiabetic medication prescription [2]. Approximately 90–95% of these people have type 2 diabetes [3] and the majority of them are managed in primary care [4–7].

Medical treatment and lifestyle-change, self-management are important means of achieving glycemic control and preventing complications in diabetes. However, a number of people with diabetes experience difficulties in maintaining a healthy lifestyle and adhering to medical therapy.

In their review, Rubin and co-workers reported adherence rates between 65–85% to oral antidiabetic therapy among people with type 2 diabetes [8]. In their more recent review [9], Krass et al. found little improvement over time when reviewing 27 studies: the prevalence of adherence ranged between 38.5 and 93.1%, and only 6 studies reported adherence over 80%. In line with these reviews, low persistence to certain oral antidiabetic medications has been reported in Hungary [10]. Multiple factors were proposed for these findings, depression and high healthcare costs emerging as the most important, independent predictors of suboptimal adherence [9].

Beside medication adherence, other aspects of self-management by people with diabetes play a very important role in the successful control of this chronic condition: healthy diet, physical activity, avoiding smoking and excess alcohol intake and maintaining normal weight were associated with lower mortality [11]. In a systemic review, improvement in self-management by education was associated with better glycemic control [12].

Unfortunately, adherence to healthy lifestyle is also not optimal [13]. Physicians, who wish to learn about their patients’ self-care (including medication adherence and lifestyle) to identify problem areas, might find the use of tools to assess diabetes self-management beneficial. These tools offer the possibility of obtaining objective and comparable data that is free from bias caused by different attitudes and communication skills of physicians, and are helpful in monitoring change in behaviours over time. Although there are a number of tools available for this purpose, most of them are either not validated properly, or do not correlate with glucose metabolism [14]. Effective self-management results in better glycemic control [15], decreases mortality [11] and can improve quality of life even in persons with diabetic complications [16]. Whether addressing issues of self-care that are associated with glycemic control might further improve treatment efficiency and protect the person with diabetes from late complications warrants further studies.

Schmitt et al. developed a questionnaire in German and English focusing on factors strongly correlated with HbA1c to assess behaviours associated with metabolic control in adults with type 1 and type 2 diabetes. The Diabetes Self-Management Questionnaire (DSMQ) proved to be a valid tool, which showed a strong relationship with glucose metabolism among people with mostly type 1 diabetes in tertiary care [17]. The questionnaire is available in two forms from the original authors. The 16-item DSMQ has 4 subscales: Glucose Management (GM) with 5 items, Dietary Control (DC) with 4 items, Physical Activity (PA) with 3 items and Health-Care Use (HU) with 3 items, and one general statement. The revised form (DSMQ-R), has the same structure as the original tool, but has altogether 20 statements about self-care for non-insulin-treated persons (two extra questions on diet, one on healthcare usage, a general one), and 7 added items for insulin-treated persons (20 + 7 statements).

The proportion of people with diabetes on injectable therapies has risen significantly, a large proportion of these persons being on insulin therapy [18]. Thus, the additional questions of the extended version of the DSMQ, clarifying attitudes about insulin therapy might come valuable both in daily practice and research. The revised version of the DSMQ was used in a number of studies [19–23]. The short version of the DSMQ was validated in urdu and thai languages [24, 25], and it has been recently translated to Hungarian [26], independently from our group’s work. The participants of all these studies were persons with type 2 diabetes, who were enrolled from secondary and tertiary care in two of these studies, while the exact site of enrollment was not specified in the thai publication [24].

The site of initial assessment may influence the usefulness of clinical tools in family practice. Questionnaires used in secondary-tertiary care may need further evaluation in the primary care setting, therefore different tools may be required to measure health outcomes [27]. This is especially true for diabetes, with striking differences in terms of the proportion of persons with type 2 diabetes, the duration of the disease, the number of complications or glycemic control [28, 29].

Our aim was to translate the 20 + 7 item DSMQ-R questionnaire to Hungarian language and to assess whether its validity and association with glycemic control is present in a Hungarian sample of predominantly type 2 diabetes persons in primary care.

## Methods

We enrolled adults from teaching practices attached to the Department of Family Medicine, Semmelweis University (38 practices, 432 adults) and to the Institute of Primary Health Care, University of Pécs (5 practices, 60 adults) between January 1, 2017 and February 28, 2019. Leaders of practices voluntarily agreed to take part in the study. They approached their patients with diabetes mellitus of any type and asked them to participate in the study. Physicians were required to enroll eligible persons during the first three months after agreeing to take part in the study. Adults not being able to fill out the questionnaire due to mental or vision difficulties were excluded.

The self-administered questionnaire used in this study consisted of the Diabetes Self-Management Questionnaire (DSMQ-R) and the Summary of Diabetes Self-Care Activities (SDSCA) questionnaires. Answers for the DSMQ-R questionnaire are given on a 4-point Likert scale. The questionnaire contains both positively and negatively worded statements. Scale scores are calculated by dividing the actual sum of items given by the person by the maximum possible sum of items, and then multiplying it by 10. The answers for the negatively phrased statements should be inverted.

Doctors provided clinical data about their patients by filling out the appropriate form (gender, age, BMI, type, duration and complications of diabetes, current diabetes therapy and the results of the last routine laboratory tests). Patient-level data was available only to their family physicians. Questionnaires and clinical data sheets were marked by patient code numbers and further analyzed at the Department of Family Medicine by the investigators. All persons gave informed consent.

Based on the prevalence of OAD prescriptions filled (7.27%) and the population size of Hungary (app. 9,770,000), the investigated population size was 710,279 persons. At a margin of error of 5% and a confidence level of 95% the calculated sample size was 384 [30], thus the number of persons enrolled was adequate.

### Translation of the questionnaire

Two translators (KV and BM) provided the initial, independent translations of the DSMQ-R (T1 and T2). A recording observer (Peter Torzsa) and the two translators synthesized the initial translation into the “version 1” Hungarian translation of the questionnaire. Two translators (BI and Éva Palik), blinded to the original questionnaire, provided the back-translation of “version 1”. An expert committee composed of LK and all the translators agreed by consensus on the “version 2” of the translated questionnaire, after comparing the back-translation with the original DSMQ-R. This pre-final questionnaire was tested in one practice, on a group of 35 adults to make sure that statements were comprehensible, no difficulties or misunderstandings were raised when answering them. After minor modifications following this testing, the “version 3” questionnaire was achieved. The expert committee reviewed information gathered during the translation process and approved “version 3” as the final Hungarian translation of the DSMQ-R.

We encountered no major difficulties during the forward translation of the DSMQ-R questionnaire. The few differences between the T1 and T2 translations were resolved during the synthesis of the two translations. The observed differences included semantic and sentence structure differences and the alternative use of articles. The backward translations were conducted without any notable problems, and the obtained versions were substantially very similar to the original version. During testing of version 2, none of the 35 adults included in the study expressed any difficulties understanding the questionnaire. As a final step, all members of the Expert Committee gave their approval after expressing their satisfaction with the final version of DSMQ-R.

### Validation process

#### Reliability analysis

##### Internal consistency

We assessed the internal consistency of the questionnaire by calculating the Cronbach’s alpha coefficient for each subscale and for the sum scale, and by investigating inter-item and item-total correlations and the effect of item deletion on the coefficient.

#### Construct validity

We evaluated the construct validity of the questionnaire by analyzing known-group validity and convergent validity.

##### Known-group validity

We analyzed known-group validity by assigning adults to the on target and not on target groups (HbA1c ≤7.0% or > 7.0%, respectively), based on the ADA and Hungarian guidelines, that recommend this glycemic goal for most people with diabetes [31, 32]. Patients’ self-care activities, measured by the DSMQ-R questionnaire were compared between the two groups.

##### Convergent validity

We investigated the correlation between the DSMQ-R and the SDCA questionnaires and their matching subscales, to assess convergent validity. We also analyzed the relationship between DSMQ-R results and clinical outcomes representing the quality of diabetes care (HbA1c, BMI).

### Statistical analysis

Statistical analysis was performed with SPSS v25 (SPSS, Chicago, IL, USA). *P*-values <0.05 were considered statistically significant. We used the Mann Whitney U test for group comparisons. Since none of the variables were normally distributed, we used Spearman’s correlations. Multivariate logistic regression analysis was used to analyze the association of the DSMQ-R score and other patient variables with glucose metabolism.

## Results

Family physicians approached five hundred and seventeen eligible adults. Two persons were excluded due to vision difficulties, twenty-three persons declined participation (11 for not having enough time, 12 adults not providing specific explanation). The data of 492 persons were further analyzed. Main characteristics of enrolled persons are summarized in Table 1.

**Table 1:** Characteristics of adults filling out the Hungarian version of the Diabetes Self-Management Questionnaire in primary care

**Gender (%)**	
Males	237 (48.2%)
Females	255 (51.8%)
**Age (years)**	65.4 ± 11.7
**BMI (kg/m**^**2**^**)**	29.8 ± 5.9
**Type of diabetes (%)**	
Type 1 diabetes	27 (5.5%)
Type 2 diabetes	465 (94.5%)
**Duration of diabetes (years)**	12.4 ± 9.7
**HbA1c (%)**	7.14 ± 1.38
**Diabetes therapy**	
**Noninsulin therapy**	
Metformin	358 (72.8%)
Sulfonylurea and glinides	108 (21.9%)
Acarbose	10 (2.0%)
Pioglitazone	1 (0.2%)
DPP-4 inhibitor	69 (14.0%)
SGLT-2 inhibitor	26 (5.3%)
GLP-1 receptor antagonist	16 (3.3%)
**Insulin therapy**	
Basal or premix insulin	75 (15.2%)
Insulin intensive conservative therapy	110 (22.4%)
**Intensity of diabetes therapy**	
No medical therapy (beyond lifestyle advice)	16 (3.3%)
1, OAD monotherapy (metformin)	174 (35.4%)
2, Dual therapy (metformin + other oral agent with intermediate efficacy)	39 (7.9%)
3, Dual therapy (metformin + other noninsulin agent with high efficacy)	48 (9.8%)
4, Triple therapy without insulin	30 (6.1%)
5, Basal or premix insulin (± other noninsulin agents)	75 (15.2%)
6, Insulin intensive conservative therapy (± other noninsulin agents)	110 (22.4%)
**Late complications of diabetes (%)**	
With late complication	199 (40.45%)
Number of complications per affected person	1.73
**Detailed late complications of diabetes (%)**	
Retinopathy	75 (15.2%)
Nephropathy	74 (15.0%)
Neuropathy	105 (21.3%)
Diabetic foot	43 (8.7%)
**Cardiovascular disease (%)**	222 (45.1%)
**DSMQ points**	
Sum Scale	7.08 ± 1.52
Glucose management subscale (GM)	7.26 ± 1.87
Dietary control subscale (DC)	6.80 ± 1.99
Physical activity subscale (PA)	6.12 ± 2.87
Healthcare use subscale (HU)	7.99 ± 1.91

Continuous variables are given as mean ± SD, categorical variables as number and percentage

Treatment intensity was classified based on the number of medication used to control diabetes, and the glucose lowering effect of the agent used, as categorized Table 9.1 of the ADA guideline [33] (I: Monotherapy; II: Double combination of metformin and an agent with intermediate efficacy (acarbose, DPP-4 inhibitor or SGLT-2 inhibitor); III: Double combination of metformin and an agent with high efficacy (sulfonylurea, glinides, pioglitazone or a GLP-1 agonist); IV: Triple combination of noninsulin agents; V: Insulin once or twice daily; VI: Insulin more than twice daily).

### Reliability analysis

#### Internal consistency

We computed Cronbach’s alpha for the DSMQ-R sum scale and its subscales. The reliability analysis was carried out on the sum scale comprising 20 items (statements on insulin treatment were analyzed as a subscale). Cronbach’s alpha showed the questionnaire to reach good reliability (α = 0.845). Most items appeared to be worthy of retention, resulting in a decrease in the alpha if deleted. The one exception was item 15 (“I am less physically active …” ), which would minimally increase the alpha to 0.846.

The glucose management subscale (GM) consisted of 5 items, and the reliability was questionable (α = 0.634). Statement 12 (“I tend to forget or skip my diabetes medication”) showed exceptionally low item-total correlation (0.107), and the removal of this statements would increase Cronbach’s alpha (α = 0.688). This subscale had 11 items for adults on insulin therapy. Among these 185 persons the reliability of the subscale was acceptable (α = 0.766), but the removal of statement 12 would result in an increase of the alpha (α = 0.776).

The dietary control subscale (DC) comprised 6 items (7 for persons on insulin treatment), showing acceptable internal consistency (α = 0.709). The removal of any of its statements did not increase Cronbach’s alpha.

The physical activity subscale (PA) consisted of 3 items and had acceptable reliability (α = 0.774). All three items were worthy for retention.

The health care use subscale (HU) comprised 4 items, and its reliability was poor (α = 0.539). Statement 19 (“I get my doctor’s / nurse’s / health professional’s opinion to adjust/ optimize my diabetes treatment”) had a low item-total correlation (0.158), and it was the only statement that, if removed, would increase the Cronbach’s alpha (α = 0,589).

#### Construct validity

##### Known-groups validity

Based on their HbA1c values 269 adults belonged to the controlled, and 223 to the uncontrolled group. The mean interval between completing the DSMQ-R questionnaire and the last HbA1c result was 85 ± 121 days (mean ± SD). The controlled group had significantly higher scores on the DSMQ-R sum scale (median (interquartile range): 7.23 (6.17–8.44) vs 6.91 (5.91–8.02), effect size: 0.102, *p* = 0.027). However, among the subscales we only found marginally significant difference on the Glucose management subscale (median (interquartile range): 7.33 (6.00–8.79) vs 7.30 (5.83–8.48), effect size: 0,098, *p* = 0.033).

We found no significant difference between the controlled and uncontrolled groups in the SDSCA sum scale, or in any of its subscale scores (data not shown).

##### Convergent validity

We assessed the correlations between the DSMQ-R and the SDSCA questionnaires and external criteria associated with diabetes care (Table 2).

**Table 2:** Spearman’s correlation between the Diabetes Self-Management Questionnaire scales and BMI, HbA1c and Summary of Diabetes Self-Care Activities scales

DSMQ					
	Glucose management	Dietary control	Physical activity	Healthcare use	Sum Scale
BMI	−0.051	−0.128**	−0.209**	0.002	−0.155**
HbA1c	−0.069	−0.069	0.007	0.022	−0.095*
SDSCA blood-glucose testing	0.327**	0.316**	−0.023	0.262**	0.339**
SDSCA general diet	0.310**	0.568**	0.146**	0.315**	0.513**
SDSCA specific diet	−0.055	−0.052	0.038	0.011	−0.024
SDSCA exercise	0.117**	0.177**	0.575**	0.147**	0.328**
SDSCA foot care	0.145**	0.232**	0.122**	0.190**	0.252**
SDSCA smoking	0.007	0.015	−0.020	−0.073	−0.026
SDSCA sum scale	0.323**	0.454**	0.290**	0.357**	0.520**

Coefficients printed in bold represent convergent validity. * *p* < 0.05, ** *p* < 0.01

The DSMQ-R sum scale showed a weak correlation with HbA1c levels (*ρ* = −0.095, *p* < 0.05). In partial correlation the correlation between the DSMQ-R sum scale and HbA1c increased to 0.123 (*p* = 0.008) after controlling for intensity of therapy. Age and HbA1c were negatively correlated (*ρ* = −0.151, *p* = 0.001). The SDSCA sum scale and its subscales however, did not correlate with HbA1c values (data not shown).

We performed regression analysis to account for factors influencing effectiveness of care (Table 3). Late complications were the sum of the presence of retinopathy, nephropathy, neuropathy, atherosclerosis and diabetic foot. In multivariate logistic regression analysis, the sum scale of the DSMQ-R, age, and most prominently the intensity of therapy were significant determinants of having controlled diabetes. The model was statistically significant (χ2(6) = 55.437, *p* < 0.001), explained 15.0% (Nagelkerke R^2^) of the variance in diabetes control and correctly classified 63.2% of cases.

**Table 3:** Determinants of achieving target goal (HbA1c < 7.0%, logistic regression analysis) among primary care patients

Variable	OR	CI 95%	***p***
**Age**	1.027	1.008–1.046	0.005
**Gender**	1.307	0.882–1.936	0.181
**BMI**	977	0.944–1.011	0.186
**Intensity of therapy**	0.773	0.699–0.855	< 0.001
**Number of late complications**	0.875	0.724–1.057	0.166
**DSMQ-R sum score**	1.201	1.047–1.346	0.009

The DSMQ-R and the SDSCA sum scales showed moderate, but strongly significant correlations. The related subscales had the strongest associations (Glucose management – Blood glucose testing, Dietary control – General Diet, Physical activity – Exercise).

## Discussion

The Hungarian language version of the DSMQ-R questionnaire proved to be a comprehensible, valid and reliable tool to assess the self-management of people with diabetes in primary care. The number of available tools to evaluate self-care has been limited in Hungary so far. There is only one questionnaire in Hungarian, that also evaluates self-management [34]. However, it is focused on adherence and was validated among children and adolescents with T1DM. Recently, the Hungarian translation of the short form of the DSMQ has been validated in a smaller sample of secondary, tertiary care patients [26]. From the point of view of primary care, one of the weaknesses of the validation process of the original questionnaire was the population in which it was evaluated. Adults in the original study were inpatients in a tertiary referral diabetes center, with equal percentages of people with type 1 and 2 diabetes. This design may limit its generalizability, especially for primary care, where most adults have T2DM, shorter duration of diabetes, less microvascular complications, and better glycemic control [28, 29]. Indeed, there were considerable differences between the original and our study populations. The duration of diabetes was shorter in our study (12.36 ± 9.74 years vs 17.5 ± 10.4 years) and primary care patients had lower HbA1c levels (7.14 ± 1.38 vs 8.6 ± 1.5%). Among adults with T2DM (who comprised the majority of our sample) the number of late complications (40.45% vs 50.6%) and insulin use (37.6% vs 81.9%) were also lower. Despite all these differences, in our more general sample – characteristic of primary care – the DSMQ-R proved to be a valid and reliable tool.

During the translation and adaptation process we experienced no major difficulties, and we consider the final DSMQ-R equivalent to the original English version. The minor semantic or sentence structure differences were easily overcome during the synthesis of the translations. Our data indicate that the Hungarian translation of the questionnaire proved to be an understandable, clear and easily applicable tool to assess patients’ self-management.

The reliability analysis showed good internal consistency for the questionnaire, without the presence of redundancy in the questionnaire. The glucose management and healthcare use subscales however, had lower alphas. In the GM subscale the lower agreement was mainly caused by Q12 (“I tend to forget or skip my diabetes medication …” ). Based on the answers, the majority of people seldom skip or forget their medication. However, the one-year persistence of noninsulin medications ranged between 47.0–75.6% in one Hungarian study [10], being as low as 30% for metformin in another one [35]. These reports contradict the notion of persons constantly taking their medications meticulously. In the HU subscale Q19 stood out (“I get my provider’s opinion on treatment …” ), with generally low agreements on the Likert scale. Patients seemed to be inactive about asking their doctors, nurses for opinion and advice on diabetes treatment. This sort of inactivity (Q19) and the avoidance of admitting that they did not follow doctor’s instructions regarding diabetes therapy (Q12), is probably a cultural issue. According to the Eurobarometer report on patient involvement, the traditional doctor-patient relationship is frequently associated with communication barriers. In Central- and Eastern-European countries, there “tended to be a less balanced relationship between doctors and patients, … and there was more reluctance to have a more interactive relationship with their healthcare providers” [36]. These differences might be accountable for the unexpected answer patterns on statement 12 and 19, weakening reliability.

The DSMQ-R questionnaire scales were associated with HbA1c level, BMI and the SDSCA questionnaire scales in our study. These correlations, however, were generally weaker than those in the study of Schmitt and co-workers [17]. The known group analysis showed significant, but moderately higher DSMQ-R scores among adults with HbA1c levels not exceeding 7.0%, indicating that better self-care is associated with control of diabetes. Among the subscales though, only the Glucose management scale differed significantly. In convergent validity the sum scale of the DSMQ-R questionnaire correlated weakly with HbA1c levels, although after correcting for intensity of therapy the correlation strengthened. In multivariate analysis this relationship remained significant, and the odds ratio of the DSMQ-R score was comparable to the ORs of other significant determinants. We found stronger associations between the sum scale, the Dietary control and the Physical activity subscales with BMI, a causal and aggravating factor in diabetes.

People with type 2 diabetes mellitus form a heterogeneous group, with numerous factors beyond self-management influencing diabetes outcomes. In the complex interplay of patient-, disease-, treatment-related variables no single factor seemed to stand out. While the relationship between self-care measured by DSMQ-R and glycemic control could be low among all persons with diabetes in primary care, it might help to identify those persons who are falling behind in self-management, and thus clinically meaningful improvement could be achieved in daily practice. Weaker association between the DSMQ-R and HbA1c concentrations might also be related to other factors affecting the efficiency of treatment, such as: intensity and modality of the therapy, clinical inertia, or quality and availability of formal education. For people with diabetes on insulin treatment radical changes in therapy are not achievable. Even switching to analogue insulin yields little benefit in terms of HbA1c improvement [37]. Among these people self-management is crucial in diabetes outcomes. However, switching a person with type 2 diabetes to a GLP1RA - basal insulin analog combination could bring significant improvement in control [38], despite unchanged or weak self-management activities. Clinical inertia often hampers effective treatment in the early stages of T2DM, and it increases as the number of antidiabetic medications rises [39]. In our study younger age was negatively associated with glycemic control, supporting that this delay in therapy modification was affecting our sample, too. Clinical inertia could lead to elevated HbA1c levels despite active self-management. While practically all people with type 1 diabetes receive formal education, the proportion of persons with type 2 diabetes referred to such programs could be as low as 35% [40]. Some persons with type 2 diabetes might strive for effective self-management (and score high on the DSMQ-R), but without proper knowledge gained through structured education, they are likely to fail in achieving good diabetes outcomes. All these confounding factors affect primary care patients with diabetes, which can explain the weaker association between self-management and glycemic control.

The DSMQ-R and SDSCA sum and subscales correlated strongly between parallel measures supporting validity. The SDSCA scores did not differ between the controlled and uncontrolled groups, and we found no correlation between SDSCA scores and HbA1c levels, either. In situations when there is a clinical or research need for measuring the association between self-management and glycemic control, the DSMQ-R questionnaire might be superior to the SDSCA questionnaire. On the other hand, the SDSCA contains only 11 statements [41], while the DSMQ-R comprises 20 statements (plus 7 for insulin treated persons), making its use more difficult in daily care. However, patients could quickly fill out both self-administered questionnaires outside the office, without further increasing time-pressure during the consultation. The additional items of the DSMQ-R provide more detailed information about self-care activities, although data about smoking habits and foot care needs to be gathered separately. Health-care usage and aspects of insulin treatment are covered only by the DSMQ-R. The two questionnaires use substantially different time spans: the SDSCA gathers information about the past 7 days of self-care, while the DSMQ-R covers 2 months. The wider time frame could be useful in the reliable assessment of self-care and could contribute to the better prediction of glycemic control.

### Strength and limitations

The cross-sectional nature of our study is a limitation, as HbA1c levels at a certain date might not accurately represent the glycemic control. Change of self-care over time or the intensity of therapy could lead to variations in HbA1c level, especially in type 2 diabetes where substantial differences in the efficacy of certain treatment modalities exist. A test-retest design could have provided further data about the reliability of the questionnaire.

As discussed above, the association between DSMQ-R scores and glycemic control was weak. The differences in treatment modalities and formal education in a population consisting of predominantly persons with type 2 diabetes could explain this finding, however, this relationship needs further confirmation.

Similarly to the original study, we enrolled patients with both type 1 and type 2 diabetes. In line with literature, the number of people presenting with T1DM in primary care was low (5.5%), making it unreasonable to separately analyze and report their data. We still checked whether the answers of adults with T1DM might have biased our results (data not shown). We found no difference between the type 1 and type 2 diabetes groups in terms of the DSMQ-R sum or subscale scores, in HbA1c concentrations or in the percentage of persons with good glycemic control (Mann Whitney and Khi square tests). Thus, the small number of answers from the type 1 diabetes group are very unlikely to have changed the overall results.

We consider the two statements that weakened internal consistency, a representation of a slightly more paternal doctor-patient relationship and patient inactivity, which might limit the generalizability of our results in a more patient empowering environment. However, the presence of a more balanced relationship would make these statements stand out less, thus probably would even improve internal consistency.

We consider a strength of our study that the sample size was relatively large, and fairly typical of primary care populations [28].

## Conclusion

The Hungarian translation of the DSMQ-R is a comprehensible tool to assess the self-management of people with diabetes for clinical evaluation or for research. The questionnaire is a valid and reliable tool in family practice, although its association with glycemic level is weaker in primary than in tertiary care.

## Data Availability

The datasets used and/or analyzed during the current study available from the corresponding author on reasonable request.
